# The squirrel barrier concept and its bearing on the evolution of aerial mammals

**DOI:** 10.1186/s12862-026-02532-w

**Published:** 2026-05-29

**Authors:** Norberto P. Giannini, Alan Cannell

**Affiliations:** 1 Lillo Executive Unit, National Scientific and Technical Research Council (CONICET), Miguel Lillo Foundation, San Miguel de Tucumán, Tucumán Argentina; 2 Faculty of Natural Sciences and Miguel Lillo Institute, National University of Tucumán, San Miguel de Tucumán, Tucumán Argentina; 3 Department of Mammalogy, American Museum of Natural History, New York, USA; 4 Italian Institute of Human Paleontology, Rome, Italy; 5 University of São Paulo, São Paulo, Brazil

**Keywords:** Gliding, Powered flight, Cynocephalidae, Colugo, Flight transition

## Abstract

Three vertebrate lineages conquered powered fight in different epochs of the Phanerozoic: pterosaurs, dinosaurs (birds) and mammals (bats). The evolutionary mechanisms proposed for the demanding transition to powered flight have been contentious. For mammals, recent evidence suggests that the Darwinian hypothesis of a gliding transition is supported, based on aerodynamic and paleo-atmosphere reconstructions. However, it is surprising that the many (at least 12) confirmed, independent lineages of gliding mammals that existed since the Jurassic to the present never evolved powered flight. To explain this, Pennycuick in 2008 advanced the theoretical concept of the squirrel barrier: gliding mammals face a tradeoff between the cost of abandoning their agile arboreal lifestyle, and the potential gains of powered flight, thereby limiting the evolution of the higher-aspect-ratio wings required for achieving the latter. This concept was seldom discussed, used or tested. Here we examine critically the squirrel barrier concept and its implicit components, contrasting its predictions with available evidence, specifically from morphology, aerodynamics, and fossils. We found the concept highly relevant, and more complex than originally stated. A more nuanced approach to the probable conditions that must be met to transition from the locomotor style of ancestors to flying descendants reveals several intermediate such barriers, of which the squirrel barrier is one requiring profound evolutionary changes. Glide distance is a measure of glide efficiency, because the longer the glide the better the use of the large amount of energy invested in climbing up to the launch point in the canopy. Based on observational data and aerodynamics, we hypothesize that colugos (Dermoptera: Cynocephalidae) are able to maximize glide distance and glide ratio beyond average performance and thus might be beyond the squirrel barrier as proposed by Pennycuick, but still falling too short of reaching sustained powered flight, which constitute an additional, “hard” flight barrier.

## Background

Powered flight is the most demanding, yet most efficient, mode of animal locomotion [1, 2]. Fossil evidence and aerodynamic reconstructions suggest that powered flight evolved early in pterosaurs (by the Late Triassic; [3]), and at least three times in theropod dinosaurs during the Jurassic; specifically, twice in the dromaeosaurid lineage separately leading to *Microraptor* and *Rahonavis*, and another time independently in the Avialae, the lineage inclusive of birds [4]. In mammals, powered flight evolved only in bats, which originated after the K-Pg boundary in the Paleocene [5].

Recently, we showed that for bats, as first proposed by Darwin in 1859 [6], a gliding transition to powered flight is strongly supported in the hyperdense (equivalent to a sea level pressure of 1.6 KPa) paleo-atmospheric conditions reconstructed for the early Paleogene [7]. Gliding is defined as controlled descent by an organism that converts gravitational potential energy to useful aerodynamic work manifested as a directed horizontal translation (see ref [8]). The model of the archaic early Eocene bat *Onychonycteris finneyi* (Chiroptera: Onychonycteridae) is shown to be capable of both gliding and powered flight; moreover, we show that modeling the gliding *bauplan* of pro-, plagio- and uro-patagium based on the reconstructed fossil specimens, and adding an enlarging handwing of increasing aspect ratio greatly improves gliding to a point in which available muscle power allows level flapping flight [7]. These functional morphology results are also supported by evolutionary modeling studies [9].

Remarkably, the evolutionary history of mammals shows at least 12 known independent events of gliding origination identified across the fossil record and extant clades, together spanning from the Jurassic period to the Recent (detailed in Table 1). This recurrence strongly suggests a relative ease in evolving the necessary morphofunctional characters and commanding behaviors that allow gliding in mammals. In fact, the developmental basis for patagial evolution is starting to be elucidated in some groups (marsupials) as the product of convergent genomic changes traceable to specific, upstream cis-regulatory elements in key genes, such as *Emx2* [25]. To the long list of mammalian gliders, bats may be added; following Darwin [6]. However, none of these various origins of gliding can be phylogenetically linked to bats, the only true flying mammals. As one prominent example, the phylogenetic position of colugos (Dermoptera) as the closest relatives of bats and once forming the supraordinal grouping Volitantia [26], has been solidly contradicted by both molecular [27] and total evidence [28] phylogenies. In spite of the sheer number of derived characters shared with bats (at least 17 unreversed synapomorphies from diverse organ systems; [29]), colugos instead are more closely related to primates in the Euarchontaglires [27], as opposed to bats that belong in the other large boreoeutherian clade, the Laurasiatheria [30]. Therefore, bats have no known relatives with gliding capabilities, although the earliest bats known from postcrania clearly possess winged morphologies [31], and this fact has contributed to a seemingly paradoxical scenario of many gliding originations but no known direct phylogenetic connection with the single instance of evolving flapping flight in mammals.

**Table 1:** Representative taxa by locomotion mode (terrestrial, scansorial, arborealist, or glider) in diverse mammalian groups, each including gliding species

Groups	Locomotor guild (stages)			
	Terrestrial	Scansorial	Arborealist	Glider
Mesozoic mammals				
Eutriconodonta † [10, 11]	*Yanocodon*		*Jeholodens*	*Volaticotherium*,* Argentoconodon*
Allotheria † [10, 12–14]	*Megaconus*	*Rugosodon*	*Xianshou*,* Shenshou*	*Maiopatagium*
				*Arboroharamiya*,* Vilevolodon*,* Mirusodens*
Metatheria / Marsupialia				
Stem Metatheria [10, 15]				IMG II*
Acrobatidae [16]			*Distoechurus*	*Acrobates*
Pseudocheiridae [17]	*Petropseudes*	Pseudocheirus occidentalis**	*Hemibelideus*	*Petauroides*
Petauridae [18]			*Gimnobelideus*,* Dactylopsila*	*Petaurus*
Rodentia				
Sciuridae [19]	Callosciurinae (e.g., *Lariscus*)	Xerinae (e.g., *Tamias*)	Sciurini (e.g., *Sciurus*)	Pteromyini (e.g., *Glaucomys*)
Anomaluridae [20]			*Zenkerella*	*Anomalurus*,* Idiurus*,* Anomalurops*
Gliridae [21]	*Myomimus personatus*, other	*Myomimus roachi*,* Selevinia*	*Glirulus japonica*, other	*Glirulus lissiensis* †
Eomyidae † [10]		some eomyids †	many eomyids †	*Eomys quercyi* †
Primatomorpha				
Scandentia [20, 22]		*Tupaia glis*	*Tupaia minor*	
Dermoptera [23]				*Planetetherium* †, *Cynocephalus*, *Galeopterus*
**Barrier features**				
Barrier	scansorial barrier →	arboreal barrier →	gliding barrier →	Pennycuick´s squirrel barrier →
Direction	bidirectional	bidirectional	unidirectional	unidirectional
“Strength”	soft	soft	intermediate	hard

* IMG II (“Itaboraí Metatherian Group II”) is the denomination of a fossil metatherian considered a glider in [15, 24]

** This species is predominantly arboreal but frequently uses land for transportation and feeding (see ref. 50), so considered scansorial here

**Fig. 1:**
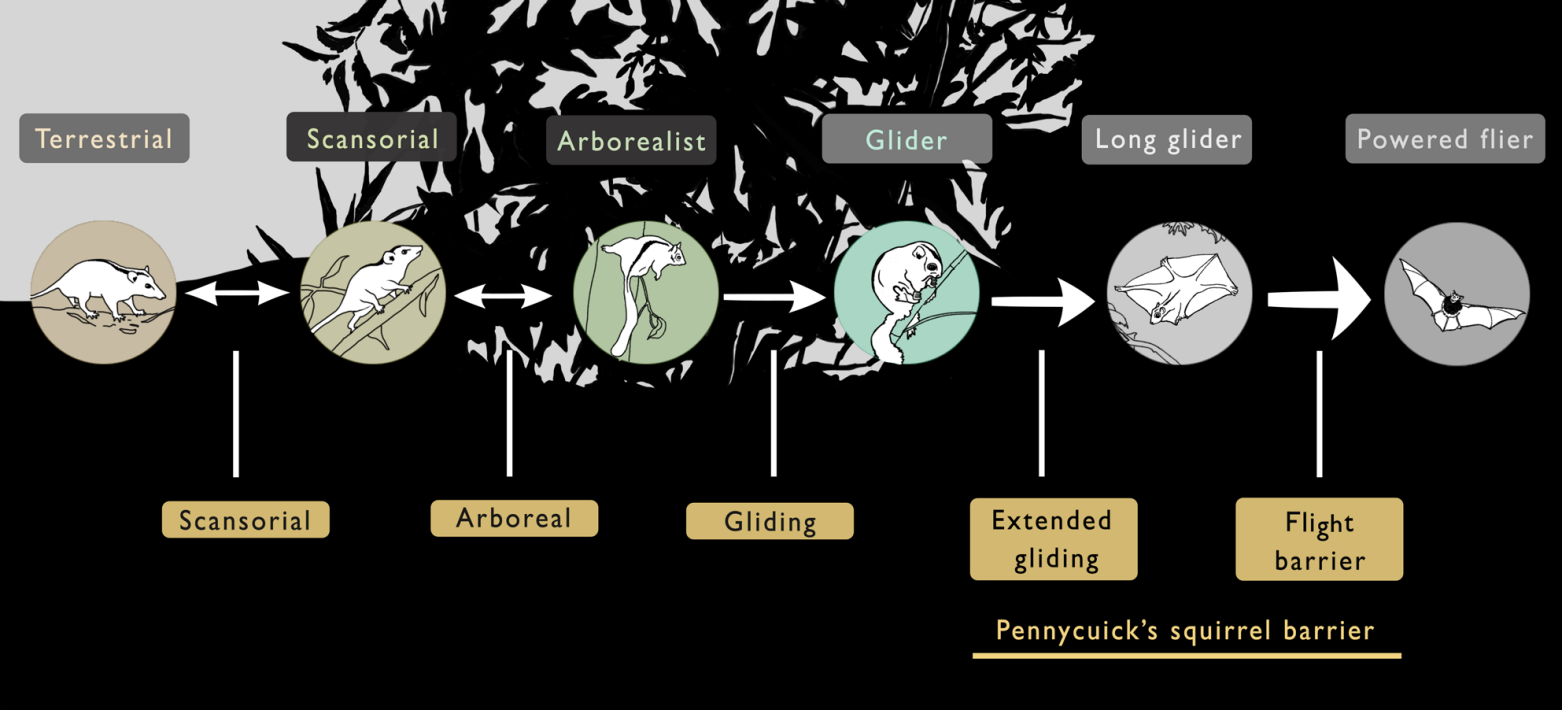
Proposed stages and barriers in a gliding transition to powered flight from a terrestrial ancestor. Stages are indicated on top of each animal figure (not to scale; figures do not represent specific taxa). Barriers are indicated with a vertical line in-between stages. Original Pennycuick´s squirrel barrier comprises here the proposed “extended gliding” and “flight barrier”. Arrows indicate proposed direction of change (bidirectional or unidirectional); thickness of the arrow suggest the relative “strength” of barriers. Colugos are in the “Long glider” stage. See text for detailed discussion. Transitions to powered flight other than the one using gliding as illustrated here may also be derived from this graph, as it is intended as a functional gradient and not as a deterministic evolutionary sequence; for instance, the “cradle-to-the-air” hypothesis [37] would be represented as a direct change from terrestrial to airborne without intermediate stages. Illustrated by Carmen Fernández de Ullivarri

Twelve independent originations of gliding appear each in one row (note that within Allotheria two separate groups are represented, *Maiopatagium* in one hand, and *Arboroharamiya* and related taxa in the other hand). The list is not exhaustive per group; single examples are given in speciose groups (e.g., Pteromyini squirrels). Extinct groups denoted †, extensive to their species; † also denotes extinct taxa within extant groups. The bottom three rows indicate features of stages represented by locomotor guild, including named barrier (e.g., Pennycuick´s squirrel barrier); if the barrier is uni- or bi-directional (i.e., if evolutionary reversals are deemed probable the barrier is bidirectional); “strength” of barrier, relative easiness for evolutionary transformation between stages (between “soft” and “hard” barriers). In this study we split Pennycuick´s squirrel barrier into the “extended glide” and “flight barrier” as shown in Fig. 1

Pennycuick [32] proposed an explanation to this paucity of flapping flight originations in the concept of the squirrel barrier, which is extensive also to pterosaurs and birds. In his words:

“Flying squirrels, and gliding arboreal mammals of other kinds, do have noticeably elongated front legs, compared with animals without patagial membranes, but the selection pressure to flatten the glides fails at the point when the limbs cannot be any longer, without impairing the squirrel’s ability to climb up trees” [32].

This squirrel barrier “limits a flying squirrel’s aspect ratio to a value well below 2, which is enough for a simple gliding wing that speeds up travel through the forest, but not enough to make level flight a practical possibility” [32]. As such, this is a highly interesting concept because it offers an evolutionary, biomechanically-based explanation to the infrequent originations of powered flight in mammals, and more generally in vertebrates, in spite of the many gliding linages with potential for evolving such ability. However, the squirrel barrier concept has been seldom considered; apparently, only Kaiser and Dyke [33] explicitly applied it, in this case to the interpretation of flight evolution in birds and related theropods in the context of evolving lifting surfaces (patagia and feathers). Therefore, its utility, or even its scientific merit, have not been thoroughly evaluated. Yet the potential of the squirrel barrier as explanation of this seemingly paradoxical scenario in the evolution of powered flight remains.

Here we set out to investigate the structure of the concept as scientific hypothesis, and to evaluate possible ways of testing its chief prediction, including aerodynamic reconstructions of aerial capabilities of key taxa. We show that the squirrel barrier is meaningful, but more complex, and propose an extended concept of barriers that pose challenges to the evolution of aerial mammals from their non-aerial ancestors, showing that these barriers can be conceptually specified with relative precision (see ref [9]) even when they might be part of an evolutionary continuum. We empirically examine features of gliding mammals, with emphasis in colugos (Mammalia: Dermoptera), which are widely considered the most accomplished gliders [34], thereby testing the squirrel barrier on aerodynamic grounds.

## Methods

### Theoretical analysis

We examined the squirrel barrier concept as proposed by Pennycuick [32] in terms of: (1) The evolutionary processes invoked, whether explicitly or not; (2) Concept complexity, i.e., if the squirrel barrier represents a single concept or, alternatively, more aspects are conflated in the evolutionary explanation of Pennycuick; (3) Testability, specifically which aspects can be directly approached with empirical data.

For the first point, we focused on the structure of the squirrel barrier as the hypothesis by examining which gliding animals have not continued evolving in the expected direction toward powered flight; i.e., a possible reason why gliding mammals with greater limb aspect ratios would be selected against, not producing an evolutionarily increase of wing aspect ratio to achieve flapping flight. Secondly, we consider the types of processes that must be involved in order to justify, and eventually be able to test, the squirrel barrier as evolutionary concept, whether it be micro- or macro-evolutionary, and under which specific theories the squirrel barrier narrative can be framed. For the third point, we take our revised concept, which involves a refinement and subdivision of Pennycuick´s original observation (see below), and design possible research strategies to test these proposals including, for instance, phylogenetic relationships (see Table 1) and aerodynamic evidence (see next section).

### Aerodynamic analysis

An empirical test of the squirrel barrier might involve finding aerodynamic parameters that must be met for the concept to hold. Here we explore measures of glide efficiency, which should be within range of prediction of aerodynamic models.

Vertical climbing to reach a suitable launch position is strenuous in terms of energy demands [35]; gliding converts the potential energy acquired by climbing into kinetic energy so that a horizontal component of velocity emerges, enabling the glider to reach landing sites of various distances [24]. Thus, the longer the distance, the more efficient conversion of that potential energy. This is especially important considering that forests have a limited canopy height, although local topography may play a role if the landing point is downhill or elevated with respect to the launch point (see ref [36]). A related but more general measure is the glide ratio (GR), a dimensionless parameter commonly used to characterize gliding performance. Three distinct but aerodynamically equivalent definitions of glide efficiency are available [32]: (1) The glide index, as the ratio of glide distance to height loss; (2) Glide ratio, as the ratio of forward speed to sinking speed, which varies over the glide and is maximum at the best glide speed; and (3) the lift-to-drag ratio, *L* / *D*, “the” general measure of aerodynamic efficiency, also applied to powered flight. If observational data of glide efficiency conform to predictions of simulation models, gliders would comply with standard gliding theory and the squirrel barrier holds, because these models use aspect ratio as given, covering values from below 2 (as in the case of mammals [34]) to over 10 (as in large marine birds; see Introduction above).

To test these predictions based on GR in standard gliding, we applied the widely used model Flight 1.25 by Pennycuick [32] with settings for calculating glide polars in mammals as in Giannini et al. [7]. There we showed that the program’s output faithfully reproduces gliding parameters observed in nature, thereby validating the approach in representative taxa (e.g., the giant flying squirrel *Petaurista philippensis*, Sciuridae; [7]). Here we report new modeling for the colugo (Dermoptera: Cynocephalidae); widely recognized as the most accomplished among mammalian gliders [34], and which represent a strong test on the squirrel barrier. We contrasted the modeled gliding parameters of the Giant flying squirrel (as in ref [7]). and the colugo (Table 2) with empirical field data, either observational or reconstructed from accelerometer data (in ref [35]).

**Table 2:** Comparison of basic data and gliding performance between the Giant Flying squirrel *Petaurista philippensis* (Sciuridae) and the Philippine colugo *Cynocephalus volans* (Cynocephalidae) according to the aerodynamic model (glide polar) in Flight 1.25 [32]

Parameters	Petaurista	Cynocephalus
Body mass (kg)	1.6	1.3
Wing span (m)	0.80	0.75
Wing area (m^2^)	0.46	0.46
Aspect ratio	1.33	1.22*
Air speed (m / s)	5.6–8.6	6.7–11.0
Sinking speed (m / s)	2.44–3.02	2.08–2.64
Glide ratio	2.30–2.85	3.22–4.17
Lift coefficient	1.78–0.87	1.10–0.53
Circle radius at 24° bank (m)	7.85	10.3

* from Stafford et al. [37]

Values given as ranges are between parameter calculated at minimum sink and best glide, respectively. See full setup for each species in Supplemental Information

## Results and discussion

### Nature of the squirrel barrier, as per Pennycuick

Pennycuick [32] postulated that animals that do not fly must overcome three hurdles, *in the following order*, to be able to fly:

1. The ability to control spatial orientation while being free to move and rotate in three dimensions, without contact with the ground.
2. The development of a shape that gives a sufficiently high lift-to-drag ratio, embodied in a structure that is able to withstand the loads imposed by flight.
3. The development of a source of power that can be used to overcome aerodynamic drag.

To achieve stage 3 above, gliders are obliged to increase aspect ratio by elongating their forelimbs, thereby reducing climbing agility such that: “Once past the barrier, each of these groups [pterosaurs, birds and bats] evolved wings with aspect ratios of 6 and beyond, thus opening the way to level flight.” Considering the anatomy of known fossil bats (see ref [31]), the newly evolved fliers (early Paleogene bats among mammals) did lose their hypothetical abilities to climb with agility (see Discussion below). In fact, early bats show characteristics of suspensory mammals, as do modern bats [31].

The nature of the concept essentially is one of conflict between evolutionary forces of directional selection versus stabilizing selection, a scenario that shows key characteristics of evolving complex character systems, as suggested by Pavlicev & Wagner [38] in a more general context. The directional selection component is pulling towards extending the forelimbs and the patagial membranes they subtend, a characteristic of gliders as compared with non-gliding relatives [10], in order to increase aspect ratio and thereby aerodynamic efficiency [39]. The penalty of this change is a relative loss of agility (as compared with an arboreal ancestor) to move in tree tops [32]. This penalty is eluded by stabilizing selection, freezing the aforementioned changes to retain arboreal capabilities such as rapid movement in compliant branches (as described in [40]), in this way impeding the evolution of higher aspect ratio wings. These forces seem to keep aspect ratio and aerodynamic capabilities limited to gliding, while retaining climbing agility to its fullest. The squirrel barrier is therefore a hypothesis of stabilizing selection against directional selection evolving higher glide performance and eventually flapping flight, but the latter process stopped in its earliest phase.

Another evolutionary aspect suggested by the squirrel barrier is that it requires acquiring various character states in orderly succession (as in steps 1 to 3 above). Interestingly, this would create evolutionary stages as suggested in other organisms that also experience locomotor switches to different media, or incremental evolution [7]. Although the evolutionary process may be in itself a continuum, this allows the characterization of morphotypes for each stage, and therefore identification of taxa that may be allocated to those stages (see below and Table 1).

### Concept structure

As presented by Pennycuick (see above), the squirrel barrier per se is located between points 2 and 3 above, i.e., Pennycuick’s statement has a wider scope as it explicitly contains at least one other such barrier, of similar ontology, between points 1 and 2 (see above), which (to follow Pennycuick), can be called the “glider barrier”. To pass this barrier, an arboreal mammal (at point 1) should evolve a gliding wing (reaching point 2), which consist of patagia of different extension (e.g., with or without tail membrane or uropatagium; e.g. [41, 42]), and other features (e.g., with or without spurs; e.g. [43]). The evolutionary history of mammals abounds in well-documented examples (see Table 1) and they represent a remarkable fraction of mammalian diversity (roughly 1% or c. 66 species of extant mammals glide). This makes the gliding barrier a “soft” barrier (past by numerous mammalian lineages) as compared with the “hard” (only rarely past) squirrel and powered flight barriers (see Table 1, bottom panel, and Fig. 1). Yet another barrier is also implicit, and it involves the specialization of becoming an arboreal mammal, i.e., to reach point 1, which is a pre-requisite of the gliding theory of transition to powered flight, as first proposed by Darwin [6]. We suggest that this previous barrier, which can be called the arboreal barrier, is past when a scansorial mammal, i.e., a mammal capable of both terrestrial and non-specialized arboreal locomotion, becomes a specialized climber, reducing its dependence on ground-based resources (including those that provide nutrients, roosting, opportunities for socializing, etc.). This specialization requires precisely the kind of sensory and motor capabilities required by Pennycuick´s point 1 to negotiate a fully three-dimensional, discontinuous, and structurally complex space (see [40]). For instance, colugos´ eyes are oriented such that they possess the binocular vision needed for depth perception during gliding [23], a characteristic already present in many climbers.

Scansorial habits have been reconstructed for instance in the ancestral placental mammal [28], and for long this habit has been considered ancestral for mammals generally (e.g. [44]). However, recent analyses suggest that ancestral mammals were not scansorial but terrestrial [12]. Climbing up trees, even in scansorial mode, requires morphologies identified by Nations et al. [12] that are not present in stem mammals of any of the major clades, including therians. Therefore, here we can establish a scansorial barrier, which is past when a terrestrial mammal acquires morphologies and associated basic abilities to climb up trees, not yet abandoning strong ties to a terrestrial life.

Taken together, this orderly succession of stages has support from evolutionary models of appendage morphology [9]. In these scenarios, different locomotor groups occupy distinct optima in the adaptive landscape of forelimb morphology, with the arborealists + gliders optimum intermediate between those of terrestrial mammals and flyers (bats) [9]. From this morphospace perspective, barriers examined here, with their different strength and directionality (see Table 1), might represent adaptive valleys of different depths.

Consequently, we suggest that Pennycuick´s concept is richer than previously thought in that it implies a more complex scenario of stages and barriers. Because mammals in particular may have had fully terrestrial ancestors, the barriers to be crossed to become a propelled aerial animal would then be the scansorial, arboreal, gliding and the squirrel barrier, in that order, in the transformational path from ancestral locomotion (terrestrial) to flapping flight. In principle, the stages in-between these barriers would then be terrestrial, scansorial, arboreal, gliding, and flying. In Table 1 we show an attempt to allocate known taxa to these stages and show that many fossil and extant groups have representatives in each stage. In addition, we suggest properties of barriers, specifically for mammals, if they are “bidirectional” or reversible, or if they are “unidirectional” meaning that the animals in one stage might evolve toward previous stages, like losing flight abilities back to a gliding, arboreal or terrestrial stage, as is so frequent in birds but undocumented in mammals.

### Aerodynamic performance

We expected that modeled gliding performance matched empirical data for the studied species; this would mean that, on average, low-AR gliders conform to the squirrel barrier in that they remain confined to the predicted gliding regime. Simulations using the Flight 1.25 model yielded gliding parameters reported in Table 2 for the colugo. Calculated GR was in the range of 3.22–4.17 (at minimum sink and best glide, respectively; see complete results in Table 2). Physical data, as deduced from accelerometer data in Byrnes et al. [35], match extremely well the simulations using Flight. Specifically, GR estimated from mean or maximum glide and climb length, from mean and maximum summation across all glides, or calculated from mean glide angle (15°), vary in the range of 3.81–4.19, mean 3.93 or roughly around 4 (Table 3). Consequently, given that the Flight model accurately reproduces gliding performance in mammals (see also simulations for another, unrelated glider in Giannini et al. [7], aerial locomotion in this animal is on average highly consistent with standard gliding for low-AR wings and therefore, the colugo meets the expectations under the squirrel barrier concept. When compared with a similar sized glider, the colugo outperforms the squirrel *Petaurista philippensis*, for instance GR is 40 to 46% greater than in the flying squirrel *Petaurista*, which is otherwise quite similar (but not identical) in several of the considered variables (Table 2; gliding performance in both the colugo and the squirrel are accurately predicted by Pennycuick´s model).

**Table 3:** Glide index for the colugo as the ratio glide distance / climb height, which are obtained from different parameters in Byrnes et al. (ref [31]; accelerometer data)

Type of parameter	Glide distance (m)	Climb height (m)	Estimated index
Mean length ^1^	31	8.1	3.82
Maximum length ^2^	145	36	4.02
Mean accumulated length ^3^	436	115	3.79
Maximum accumulated length ^4^	1342	320	4.19
Estimate from average glide angle ^5^	7960	2090	3.81

^1^Distance and height of individual glides averaged over all recorded glides

^2^Maximum recorded value of glide distance and climb height (values not from the same glide)

^3^Average of total distance (horizontal and vertical) travelled per night by individual colugos

^4^per night by an individual colugo (ranges of 130–1342 m and 38–320 m for glide and climb distance, respectively)

^5^Mean glide angle obtained from arcsine of summation of all heights (2090 m) divided by summation of all glide distances (7960 m) = 15°

However, several individual glides reported in the literature, or deduced from accelerometer data, indicate that the colugo greatly exceeds its average GR in some cases. Individual long glides of up to 145 m have been registered using accelerometers (as calculated from Fig. 4C in Byrnes et al. [35]), which surpasses previous anecdotal record of gliding distance of 136 m [45]. In the latter, altitude lost was 12 m, which gives GR of 11; data in Byrnes et al. [35]: Fig. 4C) gives GR between 6 and 9 for registered long glides. Clearly, while the *average* performance of the colugo (Table 3) is that of a standard glider for its low-AR wing, the animal is on record and from different sources of being capable of exceptionally long glides, which has not been observed in any of the other mammalian gliders. The possibility of very long glides may have adaptive significance, even if uncommonly used in comparison with the bulk of gliding bouts. The extent of forest openness determines degrees of freedom for establishing glide paths, which is key for conservation of gliding mammals [36], but it may have also played a role in evolution of long glides in the seasonally flooded habitats of the colugo. These can be achieved by reducing drag, increasing lift, or a combination of both, although given that the body plan does not change, drag reduction or compensation seems less likely. Hence, a probable option is that the colugo increases lift by some unknown mechanism and has gone beyond the squirrel barrier as defined by the aerodynamics of Pennycuick´s model, which predicts GR at around 4 for this animal.

The second, key component of the squirrel barrier is the loss of climbing agility: to get past the squirrel barrier in evolutionary time, fitness advantages of higher wing aspect ratios exceed those of maintaining arboreal performance. Colugos typically are clumsy and laborious climbers [46]; in fact, transportation in branches is relatively slow and highly costly, to the point that gliding does not provide energetic benefits (savings) as compared with climbing and walking a comparable distance, although gliding does save considerable time [35], probably also decreasing predation risk and / or providing rapid access to otherwise less available food resources. Colugos are four-legged suspensory mammals, just as bats are hindlimb suspensory mammals, both with sophisticated tendon-locking mechanisms that allow them to hang passively (i.e., not engaging flexor muscles) from branches in the canopy [29]. These aspects combined suggest that colugos have resigned the climbing agility they might once had (as the fully arboreal sister group of primates; see ref [27]), enhancing their gliding capabilities in the process. Therefore, both bats and colugos use (or have shifted to) a suspensory framework as basis for their arboreal locomotion [47]; one Mesozoic glider (Maiopatagium: Eleutherodontidae) has been suggested also to be a four-legged suspensory mammal [48]. Therefore, although colugos never reached powered flight (i.e., not past the “hard” flight barrier) they seem to have surpassed other typical gliders in aerial performance at the cost of climbing agility by switching to suspension. This would place them beyond the squirrel barrier as originally conceived by Pennycuick, but still short of surpassing the harder flight barrier. In fact, applying Pennycuick´s model of powered flight [32] to the colugo data (see Table 2) produced no thrust nor any effective lift by flapping, with required work by muscles beyond physiological capabilities. Specifically, myofibrils work at 139 J kg^− 1^ greatly exceeded a theoretical maximum (at 57 J Kg-1), and climb rate was strongly negative at -7.22 ms^− 1^ (with zero corresponding to sustained level flight; see ref [32]).

### Overview

Here we examined the squirrel barrier concept of Pennycuick [32]; clarified the selective forces involved; identified a series of steps for mammals evolving flight from ancestral terrestrial habits, to scansorial, arborealist, gliding and flying stages, each separated by functional barriers that we describe; and finally splitting Pennycuick´s squirrel barrier into two stages, adding a “hard” flight barrier to the end of the series (Fig. 1). Colugos show an aerodynamic performance that on average fits the squirrel barrier as per Pennycuick, but they seem to be past this barrier in extended glides on record, therefore demonstrating not to be limited to the expected gliding regime limiting all other mammalian gliders. However, they fail to surpass the “hard” flying barrier as bats did, the latter remaining the only mammals capable of propelled aerial locomotion.

The concept of the squirrel barrier proposed by Pennycuick [32] involves opposing evolutionary pressures, namely directional selection toward highly efficient, high AR wings that may facilitate reaching flapping flight, versus stabilizing selection to prevent losing climbing agility and acting as a strong limit to change in the appendicular apparatus. The concept is more complex than previously thought, involving at least two such barriers, the arboreal and the squirrel barrier. If the phylogenetic scope is widened enough to include ancestor of clades that contain fliers or gliders, particularly in mammals, the transformation sequence is longer because therians (the MRCA of all extant mammalian gliders and fliers) are shown to have been fully terrestrial instead of scansorial based on skeletal evidence [12]. This adds a scansorial barrier at the beginning of the transformation sequence, and this can be extended to Mesozoic fossils outside Theria (see Table 1). On the other end of the spectrum, colugos might be past the squirrel barrier considering that, their arboreal locomotion is energetically expensive and lacks the characteristic agility of other fully arboreal mammals including their wider-clade members (Primatomorpha), thereby complying with the loss of agility required by the squirrel barrier; and that their gliding parameters, albeit on average complying with standard gliding (Table 3), appear beyond that of other gliders and the standard expectation from their low AR wings in exceptional events on record. However, colugos obviously fall short of flying capabilities, suggesting that the barrier putatively surpassed by them is intermediate between standard gliding and the hard barrier of powered flight.

The possible reasons why colugos in their long, isolated evolutionary history did not reach powered flight capability in our view fall into two chief categories, body size and development. Colugos are comparatively large (> 1 kg) arboreal mammals and this makes flying considerably more difficult to begin, while favoring rapid gliding. This tradeoff might make a transition to flight difficult if gliding is evolving more effectively at relatively large size. During the Paleogene, bat ancestors evolved flight at a much smaller size (< 50 g; see refs [49, 50]); bats the size of colugos have evolved only recently, starting to increase size some eight Ma ago [50], which suggests that only fully evolved flying mammals can sustain flight at a size comparable to that of colugos, pushing toward the size upper limit predicted by their physiology (c. 2 kg; [51]). In turn, developmental changes are required to increase AR from a presumed gliding bauplan; specifically, evolving a long hand wing (dactylopatagium) by the mechanisms of digital elongation and retention of embryonic interdigital membranes (see review in [5, 7]). Remarkably, colugos (and no other mammalian gliders) have retained interdigital membranes (through developmental mechanisms that are still unknown), and also possess fingers elongated to some degree, but the latter condition does not contribute an increase of AR with aerodynamic consequences. In bats, digital elongation is due to upregulated BMP2 protein expression in finger rays of the developing handplate, which is > 30% higher as compared with a mouse model [52], and the fingers continue to grow until adulthood, as seen in the enlarged epiphyses of metacarpal and phalanges of digits 2-to-5 of volant juveniles. Therefore, the fundamental high AR wings required for powered flight have not evolved in colugos as they have in bats through elongation of a webbed handwing, thereby bats remaining the only mammals to have conquered powered flight.

Still, many additional issues remain, and here we mention two aspects that may affect performance of gliders and flyers in their respective locomotor regimes along their evolutionary paths. First, all known mammalian gliders, extant or fossil, are predominantly phytophagous [53], including colugos [54], while flying vertebrates are animalivorous by ancestry, including bats [5, 28, 31]. Therefore, a fundamentally different trophic physiology and sensory capability must be transformed, from carbon-based to nitrogen-based diet, to derive the transition of one known form of glider into a flying mammal. Second, bats evolved powered flight in the conditions of a likely hyperdense atmosphere (up to 1.6 bar) of the early Paleogene [7]; hyperdense atmospheric conditions equivalent to c. 1.3 bar were still present in the Miocene [55], with possibly values of 1.2 bar in the Pliocene [56], before approaching normodensity (1 bar) or present conditions. Increased air density must affect positively both gliding and flying [7]; therefore, the above mentioned are the geologic time windows to be investigated that likely favored evolutionary transitions in mammals capable of aerial locomotion.

## Data Availability

All data are available in the present paper and in Giannini et al. (2024)
